# Selective Treatment of PDA in High-Risk VLBW Infants With Birth Weight ≤800 g or <27 Weeks and Short-Term Outcome: A Cohort Study

**DOI:** 10.3389/fped.2020.607772

**Published:** 2021-01-28

**Authors:** Thowfique Ibrahim, Abdul Alim Abdul Haium, Sarah Jane Tapawan, Rowena Dela Puerta, John C. Allen, Suresh Chandran, Mei Chien Chua, Victor Samuel Rajadurai

**Affiliations:** ^1^Department of Neonatology, KK Women's and Children's Hospital, Singapore, Singapore; ^2^Duke-NUS Medical School, Singapore, Singapore; ^3^Department of Neonatology, National University of Singapore, Singapore, Singapore; ^4^Lee Kong Chian School of Medicine, Singapore, Singapore

**Keywords:** PDA, VLBW, prematurity, ductal diameter, Indomethacin

## Abstract

**Background:** Patent ductus arteriosus (PDA) causing significant left to right shunt can increase key morbidities in preterm infants. Yet, treatment does not improve outcomes and spontaneous closure is the natural course of PDA. The Impact of PDA on 23–26-week gestation infants is uncertain. Selective treatment of such infants would likely balance outcomes.

**Objective:** To test the hypothesis that treatment of PDA in high-risk VLBW infants [birth weight ≤800 g or gestation <27 weeks, hemodynamically significant, ductal diameter (DD, ≥1.6 mm), and mechanical ventilation] and expectant management in low-risk infants will reduce the need for treatment and surgical ligation, without altering short term morbidities.

**Methods:** This prospective observational study was initiated subsequent to the introduction of a new treatment protocol in 2016. The 12-months before and after protocol introduction were, respectively, defined as standard and early selective treatment periods. In the early selective treatment cohort, PDA was treated with indomethacin, maximum of two courses, 1 week apart. Surgical ligation was considered after 30 days of age if indicated (DD ≥2 mm, mechanical ventilation). Primary outcomes were need for treatment and rate of ligation. Protocol compliance and secondary outcomes were documented.

**Results:** 415 infants were studied, 202 and 213 in the standard treatment and early selective treatment cohorts, respectively. Numbers treated (per protocol) in the standard treatment and early selective treatment cohorts were 27.7 and 19.3% (56/202 and 41/213) (*p* = 0.049), and the respective ligation rates were 7.54 and 2.96% (*P* = 0.045). Secondary outcomes were comparable.

**Conclusion:** The early selective treatment protocol reduced the rates of treatment and surgical ligation of PDA, without altering key morbidities. Further studies under a randomized control trial setting is warranted.

## Introduction

Patent ductus arteriosus (PDA) is a congenital cardiac condition found in 31% of very low birth weight (VLBW) infants (1). Opinion among neonatologists on how to approach the condition is divided, with treatment strategies lacking consensus (2). PDA acts as a shunt by diverting blood from systemic circulation to pulmonary circulation in preterm infants. This ductal steal phenomenon leads to complex circulatory consequences in pulmonary and systemic circulation. These hemodynamic instabilities have been postulated to cause morbidities in preterm infants in several studies (3, 4). Contrary to the expectations, closure of PDA has failed to improve key morbidities in VLBW infants as a whole, and both medical and surgical treatments have been associated with adverse effects (5). On the other hand, even if left untreated, there is usually spontaneous closure, especially in infants of higher gestational ages (6, 7). However, the impact of hemodynamically significant PDA on very high risk infants from 23 to 26 weeks of gestation could be significant due to morbidities like massive pulmonary hemorrhage and intraventricular hemorrhage (8). Current trends in PDA management indicate diminishing rates of aggressive treatment in VLBW infants with selective and delayed treatment of the condition being advocated (9), but this approach has not been methodically tested. The aim of this prospective cohort study with historical control was to evaluate the benefits and disadvantages of selectively treating high-risk infants with a significant PDA. PDA was tolerated in low-risk infants, allowing spontaneous closure, unless the infant demonstrated evidence of early organ failure such as congestive heart failure secondary to the PDA or a rising creatinine level, indicative of early kidney injury.

## Materials and Methods

This study was approved by the SingHealth Centralized Institutional Review Board (2019/CIRB 2693). All procedures performed in this study involving human participants were in accordance with the ethical standards of the institutional and national research committees and with the 1964 Helsinki declaration and its later amendments or comparable ethical standards. Informed consent was waived for all parents. This was a prospective case control study with a historical control conducted in a level III C neonatal unit of a teaching hospital. All VLBW infants born between 1 April 2016 and 31 March 2017 were included in the early selective treatment cohort. All VLBW infants born between 1 April 2015 and 31 March 2016 were included in the historical standard treatment cohort.

### Management of PDA in Early Selective Treatment Period: Protocol Design

**Figure 1:**
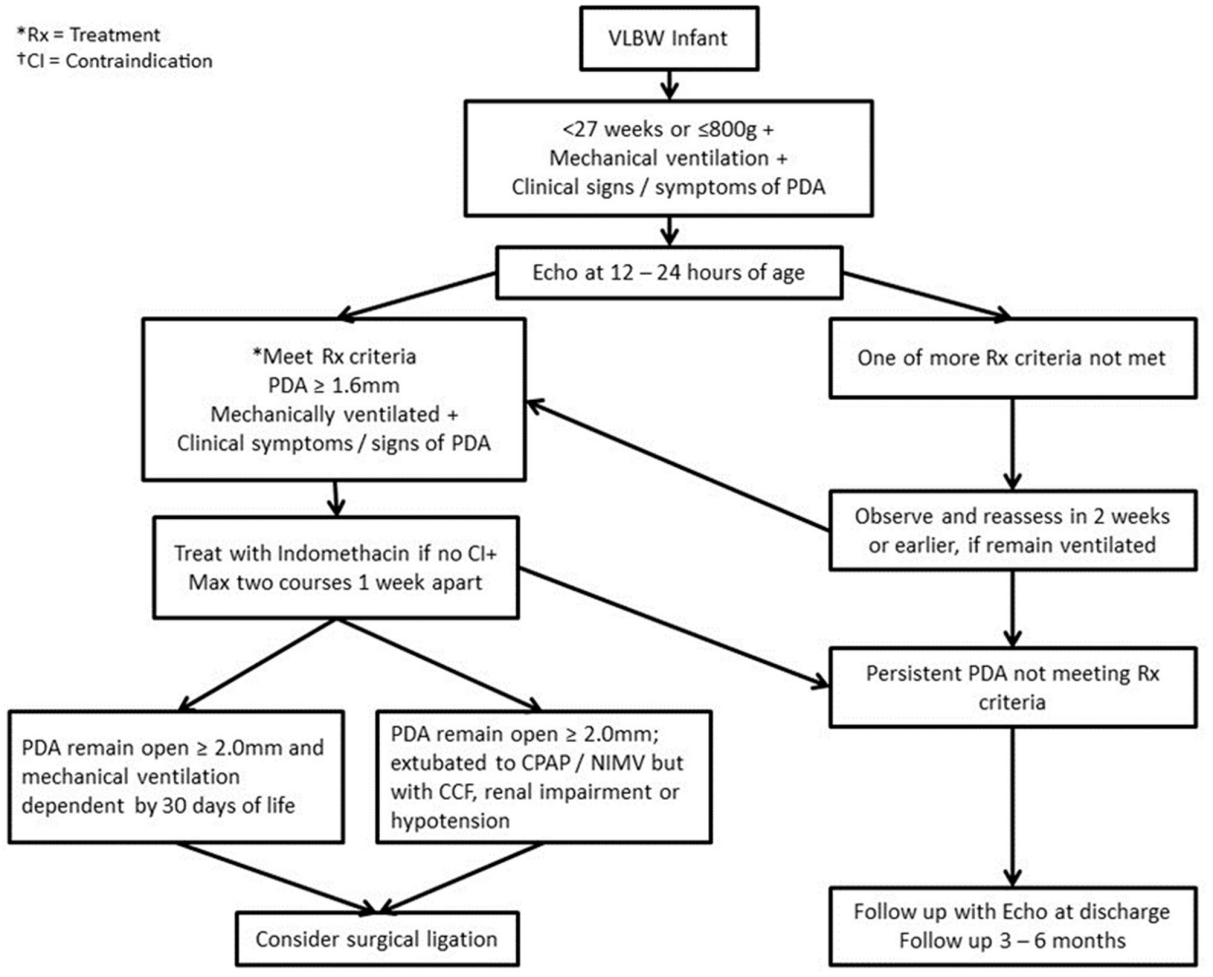
Algorithm for management of PDA in infants with birth weight ≤800 g/ <27 weeks gestation. Rx, Treatment; CI, Contraindication; CPAP, Continuous positive airway pressure; PDA, Patent ductus arteriosus; NIMV, Nasal intermittent mandatory ventilation; CCF, Congestive cardiac failure.

A consensus protocol for PDA management was prepared based on published literature, including a review article published by our department (6) with our own patient outcomes. The protocol defined screening, diagnosis, treatment, discharge and follow-up procedures for infants with a PDA (Figure 1).

### Diagnosis

All high-risk infants (high-risk group) with birth weight ≤800 g and or ≤27 weeks gestational age (GA) at birth, on mechanical ventilation and with significant clinical symptoms or signs of PDA underwent echocardiogram examination (echo) at 12–24 h of age. Significant clinical symptoms or signs were defined as follows: a grade ≥2 systolic murmur, an active precordium, hypotension, wide pulse pressure or metabolic acidosis deemed to be secondary to PDA. VLBW infants who fell outside the high-risk category (low-risk group) had an echocardiogram after 72 h of age if they were on intubated respiratory support with significant clinical symptoms or signs. For treatment purposes, a PDA was defined as significant if the infant fell into the high-risk group, required mechanical ventilation, had clinical symptoms or signs, and had a ductal diameter of ≥1.6 mm on echocardiogram.

### Management of PDA in the Early Selective Treatment Period

All the infants in high-risk group with a significant PDA were treated after 24 h of age. The main objective of the treatment was to reduce complications like pulmonary hemorrhage and intraventricular hemorrhage, apart from PDA closure. All other infants who did not belong to a high-risk group (>27 w and or >800 g) but met the criteria for significant PDA were treated at or after 14 days of life (infants between 800 and 1,000 g were considered for earlier echo and treatment at the discretion of the physician if there were significant concerns). In low-risk infants, PDA treatment was delayed to allow for spontaneous closure. VLBW infants on non-invasive breathing support were not treated unless they were showing early evidence of organ dysfunction such as congestive cardiac failure or renal impairment.

All infants with birth weight ≤1 kg, who required treatment for PDA received intravenous (IV) indomethacin. Infants with birth weight ≥1 kg were treated with oral Ibuprofen if the infant was tolerating >50% oral feeds. IV Indomethacin was preferred over IV ibuprofen because of lower cost and fewer GI complications (local experience). A maximum of two courses of indomethacin was used. All infants with a significant PDA were also treated with conservative measures, i.e., fluid restriction, for a duration at the discretion of treating physicians ( ≤130 ml/kg/day in a ≥5 days old infant). A follow-up echocardiogram was performed 72 h after completion of an Indomethacin course or on Day 7 of life, whichever was later. All high risk infants with a significant PDA and duct diameter >2 mm, despite two courses of indomethacin, were considered for surgical ligation if they required mechanical ventilation support by day 28 of life. PDA ligation was not recommended if an infant had been extubated to CPAP/NIMV. Infants whose PDA remained open underwent an echocardiogram at discharge and at 3–6 months intervals following discharge.

### IVH Prophylaxis and PDA Management

**Figure 2:**
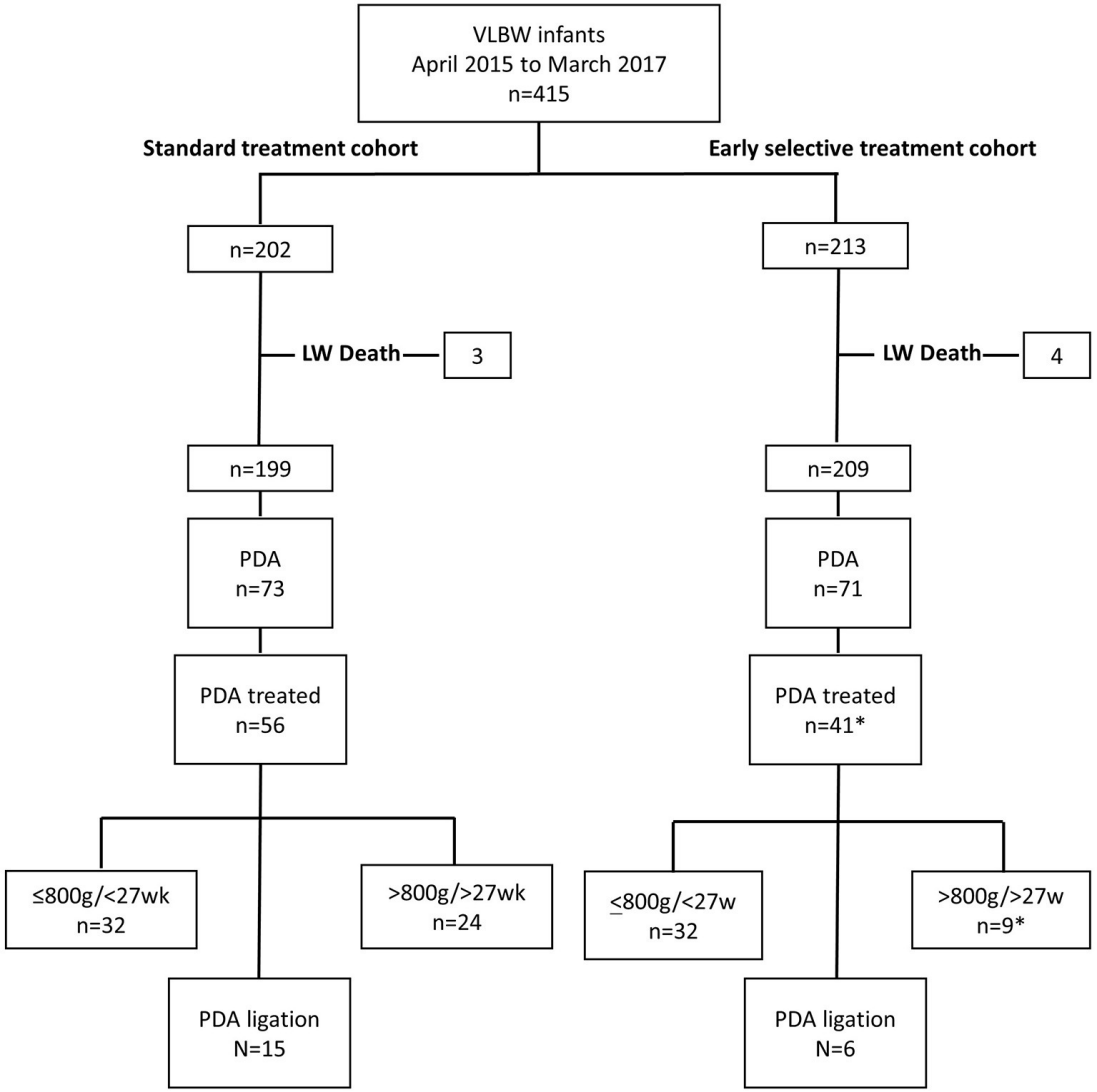
Flow diagram of study cohort representing the number of VLBW infants diagnosed with PDA, received treatment, and underwent PDA ligation in subgroups <800 g/<27w and >800 g/>27w infants in control and intervention periods, April 2015 to March 2016 and April 2016 to March 2017, respectively. LW, Labor ward; VLBW, Very low birth weight; PDA, Patent ductus arteriosus. *Excluding five infants treated out of protocol.

In accordance with the protocol implemented in April 2016, all infants born at gestational ages of ≤28 weeks or weights <1,000 g whose mothers had not received a full course of antenatal corticosteroid were given prophylactic indomethacin to prevent IVH (Figure 2, 0.1 mg/kg/day 24 h × 3 doses), with the first dose administered within 6 h of giving birth. A follow-up echocardiogram was performed 72 h following completion of treatment. If the PDA criteria for treatment were met, high-risk infants were eligible to receive one additional course of indomethacin, at least after first week of life.

### Management of PDA in the Standard Treatment Period

Treatment decisions were made on a case by case basis by individual consultants, and in doubtful cases the opinion of a senior consultant was sought before initiating treatment. Most consultants considered aggressive and early treatment in the presence of a hemodynamically significant PDA. The review manuscript (6) published by the specialists from the department prior to the introduction of the new protocol may have influenced treatment decisions. All infants with birth weight ≤1 kg, who required treatment for PDA were given intravenous (IV) indomethacin. Indomethacin doses were administered at 24 h intervals and dose varied with postnatal age of the baby. A dose schedule of 0.2, 0.1, and 0.1 mg/kg/dose was used in infants ≤48 h of age. In infants with age ≤7 days of age, a dose schedule of 0.2, 0.2, 0.2 mg/kg/dose were used. When infants were ≥7 days of age, a dose schedule of 0.2, 0.25, 0.25 mg/kg/dose were used. Infants with a birth weight ≥1 kg were treated with oral Ibuprofen if on >50% oral feeds. Three doses, at 24 h intervals were used for a course. Initial stat dose of 10 mg/kg/dose was followed by two 5 mg/kg doses.

### Data Collection

Data was collected from a prospectively maintained VLBW electronic data base, which forms part of the Vermont oxford (VON) and Australia New Zealand (ANZNN) network databases, to which our department contributes. Data collected included antenatal characteristics of the mother, delivery details, and key infant characteristics such as gestational age, key morbidities and mortality. Detailed data on PDA diagnosis, treatment and outcome of treatment were also recorded. Details of the COX-inhibitor agents used for treatment were captured for both the early selective treatment and the standard treatment cohort. Compliance data for high risk infants in the early selective treatment cohort was captured. Stratified birth weight and gestational age data were recorded for primary outcomes.

### Statistical Analysis

Data were analyzed using SAS 9.1 (SAS Institute, Cary, NC). For continuous variables, results are expressed as mean ± SD, and categorical variables were summarized as counts and percentages. Outcomes before and after instituting the protocol were compared using *t*-tests for continuous variables and Fisher's exact test for categorical variables. We adjusted for potential confounding covariates on the effect of our protocol on rate of treatment and ligation using a multivariable logistic regression model. In the stepwise algorithm used to select predictors, all non-significant predicators (*p* ≥ 0.20) were excluded from the final model. Unless otherwise stated, statistical significance was set at *p* <0.05.

## Results

**Figure 3:**
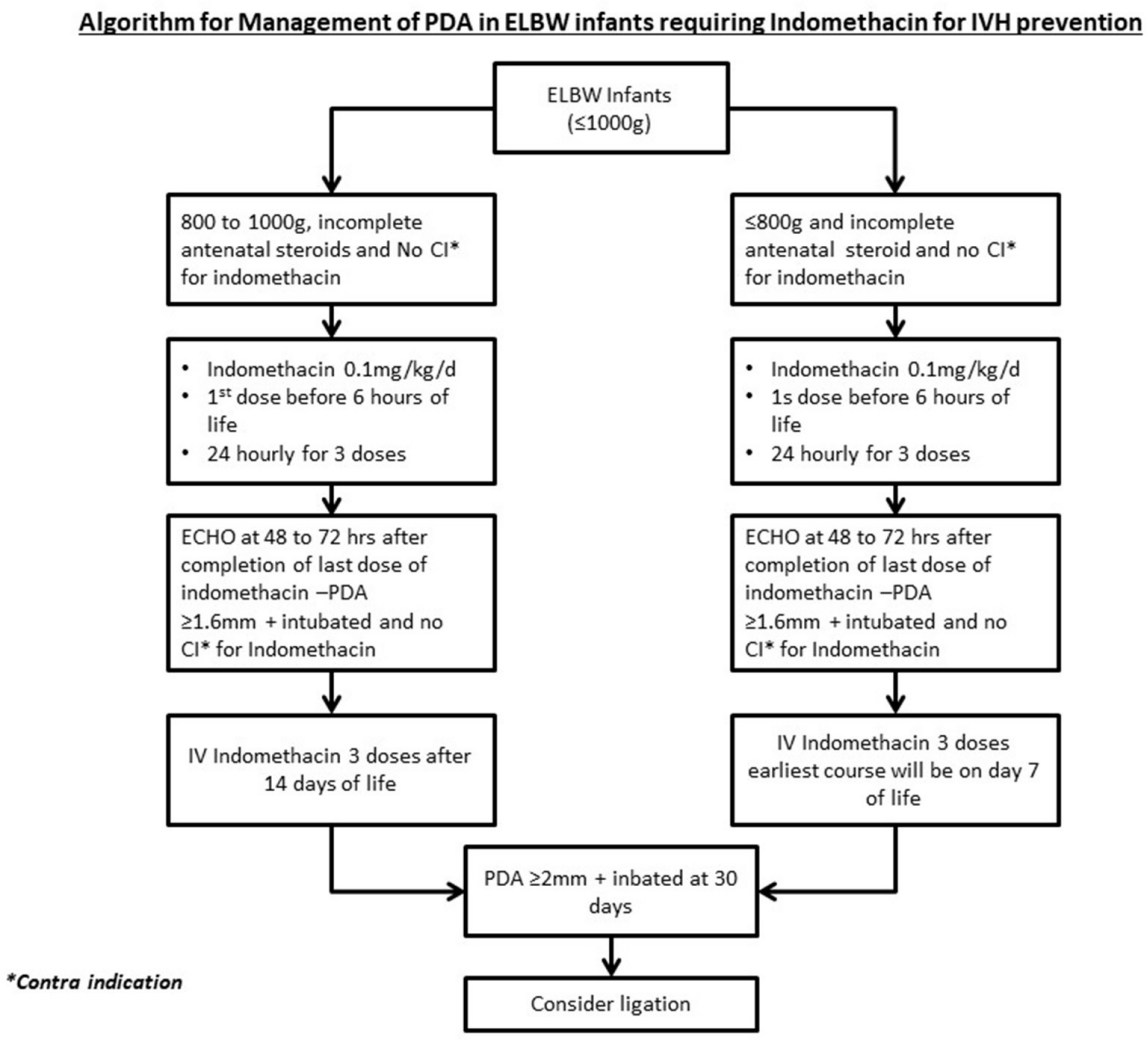
Management of PDA infants who received IVH prophylaxis. PDA, Patent ductus arteriosus; ELBW, Extreme low birth weight; IV, Intravenous.

Four hundred and fifteen VLBW infants were studied with 213 and 202 in the intervention and standard treatment cohorts, respectively. The flow diagram of subject recruitment of all 415 infants is shown in the Figure 3. Maternal and infant characteristics of early selective and standard treatment cohorts are summarized in Table 1. Comparison of primary, secondary outcomes and morbidities of the early selective treatment and standard treatment cohorts are summarized in Table 2. Stratified post-conceptional age and birth weight comparisons of treatment rates are summarized in Table 3. Detailed birth weight and post-conceptional age-based ligation rates, age at treatment, and ligation comparisons are summarized in Supplementary Tables 1–7 which are provided as Supplementary Material. Infants who received indomethacin IVH prophylaxis and subsequent PDA treatment are included in the PDA early selective treatment cohort. The percentages of infants diagnosed with a PDA was 33.3% (71/213) in the early selective treatment cohort and 36.1% (73/202) in the standard treatment group (*p* = 0.606). Indomethacin was used as the sole cox inhibitor agent in the treatment of PDA in 82.2% (37/45) of treated early selective treatment cohort and 76.8% (43/56) of treated standard treatment cohort infants, respectively (*p* = 0.624). Ibuprofen was used as the sole cox inhibitor agent in 11.1% (5/45) and 12.5% (7/56) of treated early selective treatment cohort and standard treatment cohort infants, respectively (*p* = 1.000).Three of 45 infants in the early selective treatment cohort and six of 56 infants in the standard treatment cohort received more than one COX-inhibitor agents (viz., Indomethacin, Ibuprofen or Paracetamol) (*p* = 0.513).

**Table 1:** Maternal and infant characteristics.

**Characteristic**	**Standard treatment (*n* = 202)**	**Early selective treatment (*n* = 213)**	***P*-value^a^**
Maternal age (yr), Mean (SD)	32.2 (5.76)	31.4 (5.14)	0.161
Ethnicity, *n* (%)			0.483
Chinese	104 (51.5)	102 (47.9)	
Malay	52 (25.7)	67 (31.5)	
Indian	22 (10.9)	17 (7.98)	
Other	24 (11.9)	27 (12.7)	
Pregnancy-induced hypertension (PIH), *n* (%)	45 (22.3)	52/213 (24.4)	0.643
Antenatal steroid (complete course), *n* (%)	129 (63.9)	137/213 (64.3)	0.918
Chorioamnionitis, *n* (%)	64 (31.7)	68/213 (31.9)	1.000
Mode of delivery NVD, *n* (%)	59 (29.2)	56 (26.3)	0.512
Apgar score at 5 min <6, *n* (%)	21 (10.4)	19 (8.92)	0.622
Male gender, *n* (%)	109 (54.0)	101 (47.4)	0.202
Gestational age (wk), Mean (SD)	28.8 (3.03)	29.0 (2.97)	0.547
Birth weight (g), Mean (SD)	1,055 (296)	1,072 (296)	0.556

a *Two-sample t-test (mean) or Wilcoxon rank sum test (median), Fisher's exact test (counts)*.

*IQR, Interquartile range; NVD, Normal vaginal delivery*.

**Table 2:** Comparison of primary, secondary outcomes and morbidities.

**Characteristic**	**Standard treatment (*n* = 202)**	**Early selective treatment (*n* = 213)**	***P*-value^a^**
Number infants treated with cox inhibitors, *n* (%)	56/202 (27.7)	46/213 (21.6) 41/213 (19.3)^*^	0.171 **0.049**
PDA ligation, *n* (%)	15/199^†^ (7.54)	6/203^†^ (2.96)	**0.045** 0.059^*^
Pulmonary hemorrhage, *n* (%)	4/199^†^ (2.01)	7/209^†^ (3.35)	0.544
Severe Intraventricular hemorrhage (IVH), *n* (%)	11/199^†^ (5.53)	10/203^†^ (4.93)	0.825
Spontaneous intestinal perforation (SIP), *n* (%)	3/199^†^ (1.51)	7/196^†^ (3.57)	0.337
Necrotizing Enterocolitis (NEC), *n* (%)	4/199^†^ (2.01)	3/209^†^ (1.44)	0.718
Sepsis, *n* (%)	15/199^†^ (7.43)	21/209^†^ (9.86)	0.390
Retinopathy of prematurity (ROP) stage ≥3, *n* (%)	13/185^†^ (7.03)	19/194^†^ (9.79)	0.360
Any respiratory support at 36 weeks (CLD) *n* (%)	47/185^†^ (25.4)	58/194^†^ (29.9)	0.359
Assisted Ventilation duration (days)	*n* = 197	*n* = 198	
Mean ± SD	30.3 ± 43.8	32.1 ± 45.2	0.694
Median (IQR)	12 (2, 40)	12.5 (3, 40)	0.414
CPAP duration (days)			
Mean ± SD	20.9 ± 30.1	23.5 ± 33.8	0.396
Median (IQR)	8 (1, 32)	8 (1, 33)	0.466
NICU duration (days)			
Mean ± SD	39.1 ± 42.6	47.0 ± 54.7	0.100
Median (IQR)	22.5 (12, 48)	28 (1, 33)	0.197
Death			0.554
Labor Ward, *n*	3	4	
Neonatal death, *n*	14	15	
In hospital (post-neonatal), *n*	6	10	
Total, *n* (%)	23 (11.4)	29 (13.6)	

a *Two-sample t-test (mean) or Wilcoxon rank sum test (median), Fisher's exact test (counts)*.

† *Excludes death before assessment, IQR, interquartile range*.

* *After adjusting for gestational age*.

*PDA, Patent ductus arteriosus; CPAP, Continuous positive airway pressure*.

**Table 3:** Post conceptional age and birth weight based comparison for rate of treatment.

**Low risk**				**High risk**			
**Gestation age (GA) group (mos.)**	**PDA treated control infants^a^/full-year standard cohort in Gestation age group (%)**	**PDA treated treatment infants^b^/full-year early selective treatment cohort in Gestation age group (%)**	***P*-value**	**Gestation age (GA) group (mos.)**	**PDA treated control infants/full-year standard cohort in Gestation age group (%)**	**PDA treated treatment infants/full-year early selective treatment cohort in Gestation age group (%)**	***P*-value**
≤24	15/28 (53.7)	10/21 (47.6)	0.776	>24	41/174 (23.5)	31/192 (16.1)	0.087
≤25	22/40 (55.0)	22/41 (53.6)	1.000	>25	34/162 (20.9)	19/172 (11.0)	0.016
≤26	30/56 (53.5)	29/57 (50.8)	0.851	>26	26/146 (17.8)	12/156 (7.6)	0.009
≤28	47/102 (46.0)	39/99 (39.3)	0.392	>28	9/100 (9.0)	2/144 (1.38)	0.008
≤30	55/153 (35.9)	46/156 (29.4)	0.274	>30	1/49 (2.0)	0/57 (0.0)	0.462
**Weight group (g)**				**Weight group (g)**			
≤600	6/16 (37.5)	9/16 (56.2)	0.479	>600	50/186 (26.8)	32/197 (16.2)	0.012
≤800	25/50 (50.0)	25/49 (51.0)	1.000	>800	31/152 (20.3)	16/164 (9.7)	0.007
≤1000	40/87 (45.9)	35/84 (41.1)	0.644	>1000	16/115 (13.9)	6/118 (5.08)	0.0251
≤1,200	47/91 (51.6)	41/129 (31.7)	0.003	>1200	9/111 (8.1)	0/84 (0.0)	0.011
**GA/weight group**				**GA/weight group**			
≤26/ ≤800	32/65 (49.2)	22/65 (49.2)	0.851	>26/>800	24/146 (17.5)	14/156 (9.4)	0.055

a *The numerators of the High and Low Risk PDA Treated Control Infants sum to N = 56, which is the total number of PDA Treated Control Infants; the denominators sum to N = 202, which is the number of Low Risk + High Risk Full-year Control cohort*.

b *The numerators of the High and Low Risk PDA Treated early selective treatment Infants sum to N = 41, which is the total number of PDA Treated treatment Infants (five were excluded PP); the denominators sum to N = 213, which is the number Low Risk + High Risk Full-year treatment infants*.

*PDA, Patent ductus arteriosus*.

Compliance data for high-risk infants in the early selective treatment cohort (37/45) was analyzed. There were 65 high-risk infants in the early selective treatment cohort with 31 having an echocardiogram examination at ≤24 h of age. Forty of high risk infants met the criteria for PDA treatment of which 32(80%) received treatment for PDA closure. The number of high-risk infants who received PDA closure treatment in the early selective treatment (32/65) and standard treatment (32/65) groups were identical at 49.2%. The percentage of low- risk infants who received PDA closure treatment was 9.4% (14/148) in the selective early treatment group and 17.5% (24/137) (*p* = 0.055) in the standard treatment group, respectively.

Comparison of the standard treatment cohort to the standard treatment cohort revealed no significant differences in gender, gestational age, birth weight, Apgar scores and mode of delivery, antenatal steroid use and ethnicity. Mortality, excluding labor room death, was 11.7% (25/213) and 9.9% (20/202) (*p* = 0.636) in the early selective treatment and standard treatment cohort, respectively. Mortality in treated infants was 11/41 and 4/56 in the treated and standard treatment cohorts, respectively. Causes of death in the early selective treatment group were CLD (6/11), sepsis (4/11) and pulmonary hemorrhage (1/11), and in the standard treatment group causes of death were sepsis (2/4), NEC (1/4) and CLD (1/4).

A significant reduction was observed in the number of infants requiring PDA ligation in the early selective treatment cohort compared with the standard treatment cohort, i.e., 2.9% (6/213) vs. 7.5% (15/202) (*p* = 0.042). One infant underwent ligation after discharge. The number of infants requiring PDA treatment was lower in the early selective treatment cohort [56/202 (21.6%)] than in the standard treatment cohort [46/213 (27.7%)], but the difference was not statistically significant (*p* = 0.171). Five infants received treatment outside the protocol in the early selective treatment cohort. Median (IQR) weight for infants treated outside the protocol was 1,230 g (1,018, 1,360) and gestational age 29 w + 4 d (27 w + 5 d, 30 w + 6 d). All infants were on CPAP support. After five infants were deducted from the early selective treatment group, the treatment rate was decreased to 19.3% (41/213) compared to the standard treatment 21.6% (56/202) (*p* = 0.049). The indication was borderline in another four infants for which median birth weight and gestational age were 1,290 g (950, 1,476) and 28 w + 4 d (26 w + 5 d, 30 w + 3 d), respectively. None of the latter four infants showed evidence of congestive heart failure or rising creatinine, and three were on CPAP support. There was no difference in treatment outcomes when the high-risk group was defined as ≤26 or 28 weeks. The treatment rate difference was more pronounced in low-risk infants (26/146 vs. 12/156 *p* = 0.009 in ≤26 weeks infants and 9/100 vs. 2/144 *p* = 0.008, ≤28 weeks in standard treatment and early selective treatment group, respectively). Similar outcomes were observed when the high-risk group was defined as ≤800 or 1,000 g. The median (IQR) time from birth to treatment of PDA was 67 (43, 157) and 83.5 (32.5, 288.5) (*p* = 0.804) hrs in the respective standard and early selective treatment groups. The median (IQR) postnatal age in days for PDA ligation was 36 (27, 48) and 40.5 (36, 62) (*p* = 0.352) for standard treatment and early selective treatment groups, respectively. A statistically significant difference in ligation rate was found between standard treatment and early selective treatment groups in ≤25 weeks/ ≤800 g infants.

Major morbidity rates, including solitary intestinal perforation (SIP) and intraventricular hemorrhage, did not differ significantly between the early selective treatment and standard treatment groups.11 (5.2%) infants in the early selective treatment cohort and 4 (1.88%) infants in the standard treatment cohort who received PDA treatment also received IVH prophylaxis (*p* = 0.114). 16/65 and 11/65 infants (*p* = 0.387), respectively, in the high-risk infants of earl and standard treatment cohorts received IVH prophylaxis. The incidence of pulmonary hemorrhage was comparable between groups (7 vs. 4; *p* = 0.545). Incidence of chronic lung disease (requiring supplemental Oxygen or any form of respiratory support at 36 weeks) was 29.9% (58/194) in the early selective treatment group and 25.4% (47/185) in the standard treatment group (*p* = 0.359).

## Discussion

In this study, we selectively treated VLBW infants who were at high- risk for PDA related morbidity, based on gestational age, birth weight, hemodynamic instability, PDA ductal diameter and ventilator support. Low- risk infants were treated only if they demonstrated early evidence of organ failure such as rising creatinine or congestive cardiac failure. Intervention reduced the PDA ligation rate to less than half, and reduction in treatment rate (per protocol). Results show that other key neonatal morbidities during the treatment period were comparable to the standard treatment period, thereby establishing the protocol safety. Mortality rates were comparable in the early selective and standard treatment cohorts. The authors concluded that the increase in mortality rate seen in the treated subgroup of infants was not related to the PDA protocol. Overall mortality in the VLBW infants (including labor room deaths) was reduced to 8.2% (18/219) and 8.1% (14/173) in 2018 and 2019, respectively, without modifying the protocol (10).

Treatment strategies for managing a PDA in VLBW infants vary among neonatologists and lack of uniformity is compounded by lack of agreement on the Echocardiogram characteristics that define a significant PDA (11). Management policies can be broadly summarized as three approaches, (a) expectant management with late treatment if the PDA fails to close spontaneously (b) a risk-based approach where risks are scored, tabulated and infants meeting a predefined threshold score are treated for PDA (7) and (c) conservative management defined as allowing spontaneous closure of PDA with no provision for the use of Cyclo oxygenase inhibitors (COX) or ligation. Prophylactic treatment of PDA with indomethacin or Ibuprofen lacks evidence of benefit, with near consensus on this issue in the published literature (9). The expectant approach has the disadvantage of potentially undertreating the condition, which could lead to complications such as pulmonary hemorrhage and prolonged ventilator dependency. The risk-based approach offers a more logical strategy to resolve the issue, but if not well-defined or made rigorous the process has the risk of becoming impractical. In addition, a uniform approach would be required to allow bench marking and quality assurance. With this background, we have introduced a protocol in April 2016. The primary aim of the present study was an initial assessment of this protocol. Our study provides evidence that selective treatment of PDA using a relatively simple risk-based algorithm in VLBW infants is feasible and can significantly reduce the PDA ligation rate. In addition, we found reductions in treatment rates with COX inhibitors, but no adverse impact for increasing major morbidities such as severe IVH, CLD and assisted ventilation days. The literature is divided on the issue of conservative management. A recent meta-analysis has shown no difference in morbidity or mortality when PDA is either treated with placebo or not treated (12, 13). Comparison of a large neonatal network of composite outcomes between two countries has shown a lower composite outcome defined as mortality or major morbidity with aggressive management. A safe approach is required to address this question, especially in 22- to 26-week infants (14). The outcomes of large RCTs with no treatment or placebo treatment arms—with no provision for open label treatment, is required to answer this clinical question.

Indomethacin was the therapeutic agent used for ductal closure in the majority of our infants. Moderate to low-quality evidence suggests that the efficacy of acetaminophen is equivalent to that of Indomethacin and ibuprofen, with fewer side effects (15). Nonetheless, the efficacy of paracetamol in high-risk infants is not clear, especially in infants with post-conceptional age of ≤26 weeks. Available evidence supports the use of indomethacin in high-risk infants (16). The late use of acetaminophen in persistent PDA in high-risk infants reduces the need for surgical ligation but increases the incidence of CLD and duration of respiratory support. However, acetaminophen alone or acetaminophen in combination with ibuprofen can be used as a late treatment to avoid the risk associated with surgical closure of PDA (17–19).

Our findings were comparable to those of the published literature. In a study involving 4,001 infants in a recent cohort of VLBW infants, 21.7% received intervention for a PDA in the form of either COX inhibitors or ligation. In this study the percentage of infants who underwent ligation was high, especially primary ligation (20). Although available evidence suggests improved short and long term outcomes in VLBW infants with reduced PDA treatment (21), it has not been conclusively proven, primarily due to the lack of adequate well-designed studies. Our criteria defining high- risk infants on the basis of birth weight and gestational age as essential elements is supported by the published literature (21, 23). This study combines the principle of expectant management in low-risk infants with relatively aggressive management of PDA in high-risk infants. The study used lower post-conceptional age and higher levels of respiratory support to define high-risk infants. The study also defined the criteria for identifying a small group of low-risk infants who benefit from PDA treatment. As compared to the current study, published studies testing risk-based approaches to identify infants benefitting from PDA treatment used either Echocardiogram (ECHO) based criteria alone or ECHO features combined with lower levels of respiratory support and higher gestational age to define high-risk infants (21, 24). The authors were unable to identify other published studies in the English literature that combine principles of relatively aggressive management in high-risk infants with expectant management in low-risk infants, and which incorporate defined criteria for identifying the small number of infants in the low-risk group who require treatment. Implementation of guidelines employing a conservative approach to PDA management has shown a reduction in treatment rate similar to that observed in the current study (25).

Our study has limitations. Compliance is a significant issue in the implementation of any clinical protocol. Based on the data collected, we observed 90% protocol adherence in study treatment decisions. The study is an observational study—not an RCT, and the treatment in the retrospective standard treatment group is heterogenous and not protocol based. Authors made a systematic effort to analyze the data from the period to mitigate the impact of the absence of a defined protocol in the standard treatment period. Authors have provided the additional data as Supplementary Tables that would aid sample size calculation in a future randomized controlled trial. The protocol relied on measured ductal diameter as the sole Echocardiogram feature to represent ductal significance in the treatment decision algorithm. Addition to the protocol of absence or reversal of flow in diastole would likely better characterize the magnitude of ductal shunt in VLBW infants. Following completion of this study, the IVH prophylaxis criteria were modified in our department to include only infants with GA ≤26 weeks with incomplete antenatal steroid. The number of infants who received indomethacin prophylaxis was higher (11 vs. 16) in the early selective treatment cohort. The difference was not statistically significant, although it may have some beneficial impact on the treatment rate outcome of the protocol.

Our study provides preliminary evidence that selective treatment of PDA in high risk infants is feasible without altering short term outcomes. However, the protocol needs to be validated in a large randomized control trial (RCT) with long-term follow up to obtain further evidence. Inclusion or exclusion of 26-week infants in the high- risk group is a point of contention, and inclusion with the provision of subgroup analysis is probably a pragmatic approach in designing a future study. We would consider a RCT in high- risk infants with a no treatment placebo control arm as a radical and ethically questionable approach given the current state of knowledge. The authors estimate that in a RCT setting, the need for treatment would range from 15 to 17% of VLBW infants.

## Conclusion

Our study provides preliminary evidence that selective treatment of high- risk VLBW infants with significant PDA is efficacious. Such a policy would eliminate unnecessary exposure to COX inhibitors and PDA ligation, without significantly affecting the rate of major morbidities in this vulnerable population.

## Data Availability Statement

The raw data supporting the conclusions of this article will be made available by the authors, without undue reservation.

## Ethics Statement

The studies involving human participants were reviewed and approved by SingHealth CIRB Singapore. Written informed consent for participation was not provided by the participants' legal guardians/next of kin because: exempted from written consent by the CIRB.

## Author Contributions

TI conceptualized and designed the study, drafted the initial manuscript, and reviewed and revised the manuscript. AA, ST, JA, SC, MC, and RD designed the data collection instruments, collected data, carried out the initial analyses, and reviewed and revised the manuscript. VR designed the study and critically reviewed the manuscript for important intellectual content. All authors approved the final manuscript as submitted and agree to be accountable for all aspects of the work.

## Conflict of Interest

The authors declare that the research was conducted in the absence of any commercial or financial relationships that could be construed as a potential conflict of interest.
