# Supplementary material for: Selective Treatment of PDA in High-Risk VLBW Infants With Birth Weight ≤800 g or <27 Weeks and Short-Term Outcome: A Cohort Study
Source: Front Pediatr. 2021 Jan 28;8:607772. doi: 10.3389/fped.2020.607772 (PMC7877482; doi:10.3389/fped.2020.607772)
Supplement: Supplementary file 1 [file Data_Sheet_1.docx]

Table S1. Gestation age based stratification of time to PDA treatment

| High Risk | | | | | Low Risk | | | | |
| --- | --- | --- | --- | --- | --- | --- | --- | --- | --- |
| Gestation Age Group | Standard cohort | Early selective Treatment cohort | P-value | Gestation Age Group | | standard cohort | Early selective treatment cohort | P-value |  |
| ≤24wk | 58 (36, 128) | 101 (84, 292) | 0.1792 | >24wk | | 111 ( 66, 240) | 83.5 (48.5, 307.5) | 0.2883 |  |
| ≤25wk | 60.5 (40, 98) | 84 (25, 237) | 0.4294 | >25wk | | 159.5 (72, 266) | 121.5 ( 56.5, 322.5) | 0.8649 |  |
| ≤26wk | 71 (45, 157) | 85 (32.5, 299.5) | 0.7615 | >26wk | | 162 (66, 306) | 121 ( 57, 326) | 0.8398 |  |
| ≤28wk | 92 (49, 222) | 91 (56, 307) | 0.8701 | >28wk | | 162 (56, 211) | 48 (0, 395) | 0.3092 |  |
| ≤30wk | 100 (54, 216.5) | 91 (52.5, 307.5) | 0.9763 | >30wk | | - | - | - |  |

Table S2. Weight based stratification of time to PDA treatment

| High Risk | | | | | Low Risk | | | | |
| --- | --- | --- | --- | --- | --- | --- | --- | --- | --- |
| Weight Group | Standard cohort | Early selective treatment cohort | P-value | Weight Group | | Standard cohort | Early selective treatment cohort | P-value |  |
| ≤600gm | 69 ( 25, 174) | 25 ( 22, 49) | 0.2608 | >600gm | | 102 ( 56, 222) | 126.5 ( 61.5, 314) | 0.2385 |  |
| ≤800gm | 58 ( 40, 111) | 58 (25, 219) | 0.7708 | >800gm | | 162 ( 72, 306) | 179.5 (73.5, 322.5) | 0.7110 |  |
| ≤1000gm | 85 ( 49, 184) | 84 (40, 292) | 0.8860 | >1000gm | | 173 ( 69, 286) | 234 ( 84, 395) | 0.5070 |  |
| ≤1200gm | 92 ( 49, 186) | 85 (48, 292) | 0.9155 | >1200gm | | 211 ( 89, 352) | 347 ( 84, 395) | 0.7815 |  |
| ≤ 26 weeks or ≤800gm | 67 ( 43, 157) | 83.5 (32.5, 288.5) | 0.8044 | > 26 weeks or >800gm | | 157 (67, 266) | 121.50 (58, 314) | 0.8044 |  |

Table S3. Gestation age-based stratification of rate of ligation

| High Risk | | | | Low Risk | | | |
| --- | --- | --- | --- | --- | --- | --- | --- |
| Gestation Age Group | Standard cohort | Early selective treatment cohort | P-value | Gestation Age Group | Standard cohort | Early selective treatment cohort | P-value |
| ≤24wk | 7/28(25) | 0/21(0.0) | 0.032 | >24wk | 8/174(4.5) | 6/192(3.10) | 0.587 |
| ≤25wk | 9/40(22.5) | 2/41(4.8) | 0.025 | >25wk | 6/162(3.7) | 4/172(2.3) | 0.532 |
| ≤26wk | 11/56(19.6) | 5/57(8.7) | 0.112 | >26wk | 4/146(2.7) | 1/156(0.64) | 0.201 |
| ≤28wk | 14/102(13.7) | 6/99(6.0) | 0.097 | >28wk | 1/100(1.0) | 0/144(0.69) | 0.409 |
| ≤30wk | 14/153(9.1) | 6/156(3.8) | 0.096 | >30wk | 1/49(2.0) | 0/57(0.0) | 0.462 |

Table S4. Weight based stratification of rate of ligation

| High Risk | | | | Low Risk | | | |
| --- | --- | --- | --- | --- | --- | --- | --- |
| Weight Group | Standard cohort | Early selective treatment cohort | P-value | Weight Group | Standard cohort | Early selective treatment cohort | P-value |
| ≤600gm | 4/16(25.0) | 0/16(0.0) | 0.101 | >600gm | 11/186(5.9) | 6/197(3.0) | 0.210 |
| ≤800gm | 11/50(22) | 3/49(6.1) | 0.040 | >800gm | 4/152(2.6) | 3/164(1.8) | 0.714 |
| ≤1000gm | 14/87(16.0) | 6/84(7.1) | 0.095 | >1000gm | 1/115(0.6) | 0/118(0.0) | 0.493 |
| ≤1200gm | 14/91(15.3) | 6/129(4.6) | 0.008 | >1200gm | 1/111(0.9) | 0/84(0.0) | 1.000 |
|  |  |  |  |  |  |  |  |

Table S5. Gestation age based stratification of time of ligation

| High Risk | | | | Low Risk | | | |
| --- | --- | --- | --- | --- | --- | --- | --- |
| Gestation Age Group | Standard cohort | Early selective treatment cohort | P-value | Gestation Age Group | Standard cohort | Early selective treatment cohort | P-value |
| ≤24wk | 7/28(25) | 0/21(0.0) | 0.032 | >24wk | 8/174(4.5) | 6/192(3.10) | 0.587 |
| ≤25wk | 9/40(22.5) | 2/41(4.8) | 0.025 | >25wk | 6/162(3.7) | 4/172(2.3) | 0.532 |
| ≤26wk | 11/56(19.6) | 5/57(8.7) | 0.112 | >26wk | 4/146(2.7) | 1/156(0.64) | 0.201 |
| ≤28wk | 14/102(13.7) | 6/99(6.0) | 0.097 | >28wk | 1/100(1.0) | 0/144(0.69) | 0.409 |
| ≤30wk | 14/153(9.1) | 6/156(3.8) | 0.096 | >30wk | 1/49(2.0) | 0/57(0.0) | 0.462 |

Table S6. Weight based stratification of time of ligation

| High Risk | | | | Low Risk | | | |
| --- | --- | --- | --- | --- | --- | --- | --- |
| Weight Group | Standard cohort | Early selective treatment cohort | P-value | Weight Group | Standard cohort | Early selective treatment cohort | P-value |
| ≤600gm | 4/16(25.0) | 0/16(0.0) | 0.101 | >600gm | 11/186(5.9) | 6/197(3.0) | 0.210 |
| ≤800gm | 11/50(22) | 3/49(6.1) | 0.040 | >800gm | 4/152(2.6) | 3/164(1.8) | 0.714 |
| ≤1000gm | 14/87(16.0) | 6/84(7.1) | 0.095 | >1000gm | 1/115(0.6) | 0/118(0.0) | 0.493 |
| ≤1200gm | 14/91(15.3) | 6/129(4.6) | 0.008 | >1200gm | 1/111(0.9) | 0/84(0.0) | 1.000 |

Table S7. Comparison of characteristics of PDA ligated infants

| No | Variable | Standard cohort | Early selective treatment cohort | P Value |
| --- | --- | --- | --- | --- |
| 1 | GA in weeks  Mean(SD) | 25.8(3.1) | 25(1.2) | 0.756 |
| 2 | BW in grams Mean(SD) | 736.2(212.5) | 581.6(264.6) | 0.412 |
| 3 | Age of PDA ligation in days  Median(IQR) | 36 (27,48) | 40.5(36,62) | 0.352 |
